# Retroperitoneal angiomatoid fibrous histiocytoma: A case report and review of the literature

**DOI:** 10.3892/ol.2013.1273

**Published:** 2013-03-27

**Authors:** LILI XIANG, JIAHE ZHOU, WEIYING GU, BIN YANG

**Affiliations:** 1 Department of Hematology, The First People’s Hospital of Changzhou, Third Affiliated Hospital of Suzhou University, Changzhou, Jiangsu 213003;; 2 Department of Urology, The First People’s Hospital of Suzhou, First Affiliated Hospital of Suzhou University, Suzhou, Jiangsu 215000, P.R. China

**Keywords:** angiomatoid fibrous histiocytoma, retroperitoneum

## Abstract

Angiomatoid fibrous histiocytoma (AFH) is a rare, low-grade malignant potential soft tissue tumor which occurs most commonly in children and young adults. Only a few case reports have been described that typically occur in the extremities of the deep dermis and subcutaneous tissue, followed by the trunk, as well as the head and neck. A case report of retroperitoneal AFH is described. This presentation for patients with AFH has not yet been reported. AFH may occur in the retroperitoneum, in the future patients with retroperitoneal tumor should be considered the posibility of having AFH.

## Introduction

Angiomatoid fibrous histiocytoma (AFH) is a very rare mesenchymal neoplasm of uncertain differentiation, a small number of which recur locally. Rare cases have been known to metastasize (1). It is often initially misdiagnosed as individuals often present a clinical picture resembling other diseases. AFH most often presents in children and young adults and occurs most commonly within the extremities (2–4). Previous reports have shown that AFH may occur in the extremities and the trunk. Here, a case of angiomatoid fibrous histiocytoma located in the retroperitoneum is presented. The diagnosis was established based on pathological review with immunohistochemistry. The study was approved by the Ethics Committee of Suzhou University, Suzhou, Jiangsu, China.

## Case report

A 25-year-old male was presented for evaluation with a fever and cough which had initiated 4 months previously and had become aggravated during the past 3 days. The patient noticed weight loss and denied hemoptysis, chest pain, dyspnea, fatigue or night sweats. The patient was previously healthy and had no history of smoking. His family history was negative for hereditary diseases.

On examination, the patient had a fever of 38.6°C with normal blood pressure and regular pulse. He had moderate anemia. Everything else was otherwise normal. Laboratory studies revealed anemia with a hemoglobin (Hb) of 57.9 g/l (normal adult male level 120.0–160.0 g/l), a white blood cell count (WBC) of 12.24×10^9^/l (normal level 4–10×10^9^/l) and a platelet count of 755×10^9^/l (normal level 100–300×10^9^l). His C reactive protein was 49.9 mg/l (normal level 0.0–10.0 g/l) and he had an erythrocyte sedimentation rate of 23 mm/h (normal rate 0–21 mm/h). Tests for liver function revealed severe hypoalbuminemia with an albumin level of 17.5 g/l (35.0–55.0 g/l) and a globulin level of 56.3 g/l (19.0–38.0 g/l). His prothrombin time was 18.5 sec (9.0–13.0 sec) and activated partial thromboplastin time was 41.0 sec (19.0–34.5 sec). Tests for blood and sputum culture were negative. There was no monoclonal protein on serum electrophoresis. A bone marrow blood smear demonstrated nucleated cells, myeloid and erythroid were actively hyperplastic, and a lymphocyte count of 28%. The morphology of cells were regular and thrombocytosis. Bone marrow biopsy did not demonstrate any abnormal cells. Flow cytometry of bone marrow blood did not demonstrate any monotypic cell population or increase in blast cells.

**Figure 1:**
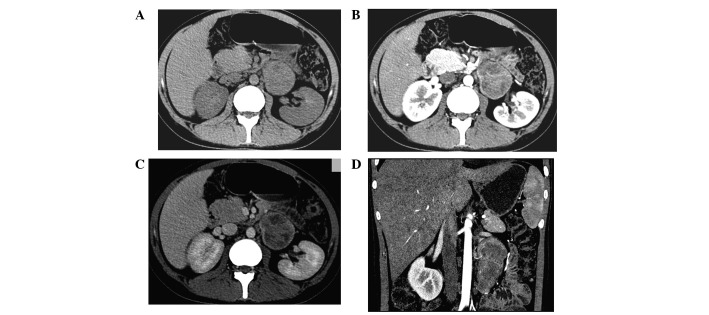
Computed tomography (CT) of the abdomen revealed an indeterminate 5.7×4.7 cm retroperitoneal soft tissue mass. (A) Precontrast; (B) arterial phase; (C) venous phase; (D) coronal arterial phase.

**Figure 2:**
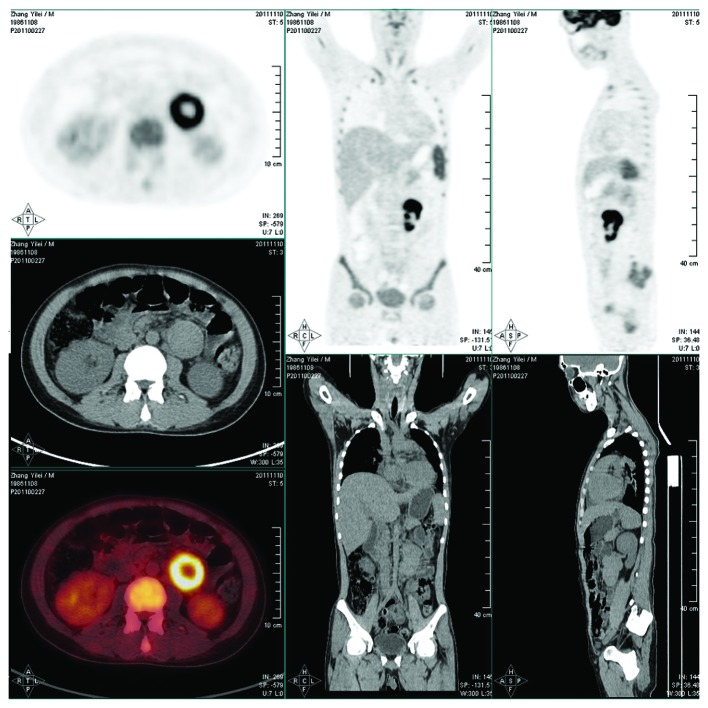
Positron emission tomography/computed tomography (PET/CT) revealed the metabolism of fludeoxyglucose (FDG) increased abnormally.

A high resolution computed tomography (HRCT) of the chest was normal. A CT scan of the abdomen showed an indeterminate 5.7×4.7-cm retroperitoneal soft tissue mass with an appearance suggestive of neurogenic tumor (Fig. 1). Positron emission tomography (PET)/CT revealed that the metabolism of fludeoxyglucose (FDG) had increased abnormally, prone to malignant disease (Fig. 2).

**Figure 3:**
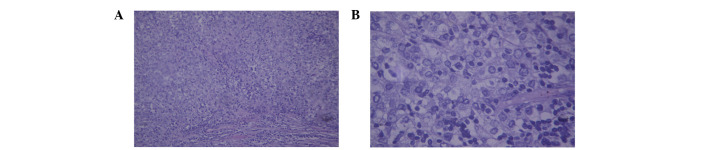
Pathological review revealed a thick fibrous pseudocapsule and lymphoplasmacytic infiltrate. Haematoxylin and eosin staining; magnification, (A) ×100; (B) ×400.

Treatment options were discussed with the patient. Retroperitoneal tumor resection was performed and a hemorrhagic firm mass measuring 8.0×5.0×5.0 cm was resected. Pathology revealed an AFH (Fig 3). Eight days after the operation, peripheral blood was Hb 120 g/l, WBC 8.20×10^9^/l and platelets 495×10^9^/l. Liver function examination showed an albumin level of 29.2 g/l and a globulin level of 44.9 g/l. Tests for coagulation function showed a prothrombin time of 15.7 sec and activated partial thromboplastin time of 37.5 sec. A month later, tests for peripheral blood, liver and coagulation function were normal. The patient had gained 5 kg in weight. Three months later his abdomen CT was normal. To date, the condition of the patient is stable.

## Discussion

AFH is a very rare mesenchymal neoplasm of uncertain differentiation, initially described as angiomatoid ‘malignant’ fibrous histiocytoma (5). It normally affects children and young adults. It typically occurs in the extremities of the deep dermis and subcutaneous tissue, followed by the trunk and the head and neck. Rare cases involve bone (6). Here, a case of retroperitoneal AFH is presented. This presentation is not unusual for patients with AFH. The clinical features of AFH may present systemic symptoms, such as fever, anemia, weight loss, polyclonal gammopathy and a Castleman disease-like lymphadenopathy (7). It is often misdiagnosed initially. A small number of AFH cases recur locally and rare cases have been known to metastasize. The best therapy for AFH is surgery together with a wide local excision. Comprehensive treatment such as radiation and chemical therapy can be used when wide excision margins are not feasible (1,8,9). According to one pathological review, AFH usually demonstrates four features: a fibrohistiocytic cell proliferation, a pseudoangiomatous pattern, a plasmalymphocytic infiltrate and a fibrous pseudocapsule (10). The immunohistochemical features present a unique immunophenotype. In one immunohistochemical review, 50–60% of cases had coexpression of desmin, epithelial membrane antigen, CD68 and CD99 but no samples were positive for CD21, CD35, clusterin or S100 (7). The diagnosis of AFH was made based on these studies. Although thorough pathologic review is critical for diagnosis, techniques such as fluorescence *in situ* hybridization (FISH) have been used to confirm cases of AFH with pleomorphic features. AFH has been found to harbor three related translocations at (12;16) (q13;p11), (12;22)(q13;q12) and (2;22)(q33;q12), resulting in an FUS/ATF1, a EWSR1/ATF1 and an EWSR1/CREB1 fusion gene, respectively (11–13).

AFH is a rare disease that occurs most commonly within the extremities and the trunk. It may also present in other parts of the body, such as the retroperitoneum in our patient. This is the first report of retroperitoneal AFH. Patients may present a clinical picture suggestive of other diseases. Pathological review is necessary to diagnose AFH. Most patients recover with wide local excision alone, but radiotherapy and chemotherapy may be utilized when wide excision margins are not feasible. AFH has a good prognosis except when it occurs in the head and neck. The recovery of our patient was good and there was no evidence of recurrence and metastasis at follow-up.
